# Ensuring sustainable mobility in urban periphery, rural areas and remote regions

**DOI:** 10.1186/s12544-023-00584-3

**Published:** 2023-04-14

**Authors:** Takeru Shibayama, Guenter Emberger

**Affiliations:** Research Unit of Transport Planning and Traffic Engineering, Institute of Transportation, TU Wien, Vienna, Austria

Transport and mobility planning has been going through a fundamental paradigm change, from conventional approaches focusing on physical and economic dimensions to minimize generalized cost of travel towards more sustainable approaches also incorporating social dimensions (e.g., Banister [2]). Regarding urban areas, the European initiative of Sustainable Urban Transport Planning (SUMP), which incorporates good practices mainly in Europe but also around the world (Rupprecht et al., [8]), is to a certain extent recognized as a state-of-the-art planning approach. In urban areas, available policy instruments for sustainable mobility are well known, and the density of cities and the concentration of knowledge and planning competence accelerate their implementation. For example, active means of transport are relatively frequently deployed as travel distances in dense urban areas tend to be short. Public transport can offer high-frequency services, and various New Mobility Services such as car sharing (cf., Shibayama & Emberger [8]) can also be introduced relatively easily due to the agglomeration of travel demand. Moreover, urban density makes it relatively straightforward to integrate land-use and transport planning, as most of the transit-oriented developments (TODs) takes place in urban areas .

However, the landscape of sustainable transport planning changes completely when it comes to urban peripheries, rural areas, and remote regions. Mobility in such areas is still predominantly supported by motorized private vehicles, whereas the existent supply of public transport and New Mobility Services are not sufficient to cover present and future travel demand. Travel distance tend to be longer compared to urban areas, and this makes the context of active modes as a mean for door-to-door travels different. Rural and remote regions often depend on a single or a few industries: the primary sector of industry is often one of the most important industries, while tourism is an industry that is increasingly gaining importance in many of such regions. Adverse socio-economic and demographic developments such as ageing, and depopulation makes it more difficult to simply apply urban approaches in these peri-urban, rural and remote regions (ITF, [8]).

Sustainable transport planning in urban peripheries, rural areas, and remote regions areas is receiving increasing attention in transport and mobility research. In parallel to our preparation of a workshop, which eventually led to this topical collection, ITF [8] gathered international experience with innovations in the field of rural mobility. In this report, ITF recommends “a profound rethink of current transport provision” in rural areas for sustainable mobility that reflects current socio-economic and demographic developments in rural areas. In transport research, intercity transport has attracted much attention; however, everyday mobility in suburban, rural and remote regions remains a rather unexplored area of transport research that receives less attention.

With this background, this topical collection (TC) is planned and realized jointly by ETRR and World Conference in Transportation Research Society’s Special Interest Group (WCTRS SIG) G2 National and Regional Transport Policy and Planning. Seven papers in this TC stem from WCTR SIG G2 Mid-term Workshop held on 27–29 September 2021. The WCTRS SIG G2 Mid-term Event was held fully online due to the COVID-19 pandemic and consequent uncertainties about international travel restrictions. It was set to cover a wide range of time zones to accommodate presenters from the Asia-Pacific Region to Americas, with 19 presentations from Australia, Japan, China, many European countries, and to Brazil. Two papers in this TC were submitted directly to ETRR in response to a specific call for papers.

Topically, the papers in this TC can be categorized into three groups. Three papers in this TC deal with policies and future scenarios of rural mobility, ranging from a national-level policy approach to local scenario building. Three papers deal with the policy instruments (measures) for sustainable rural mobility. While one deals with a concrete measure (transit-oriented development), the other two analyzes and discusses gaps between available tools and mobility needs, and implementation barriers of them. The remaining three papers deal with the topic of policy and planning of specific transport modes in rural areas, namely public transport, demand-responsive transport (DRT) with the Mobility as a Service as its variation, and regional biking.

Laa et al. [6] presents an analysis of the existing legal framework and developed scenarios for an Austrian context towards a “Nationwide Mobility Guarantee”. A Sustainable Mobility Guarantee is an approach adopted in some countries in Europe. While the paper by Laa et al. [6] is motivated by the Austrian national governmental programme of 2020, the German Federal State (Land) of Baden-Württenberg also manifested a similar concept in 2021 in its regional governmental programme. This is understood as a top-down approach to ensure a certain level of services of public transport in rural areas, demand responsive transport and other complementary modes of transport. As their research as well as the practical approaches are still in their infancies, further developments could be expected in this domain.

Nelson and Caulfield [7] analyze the impact of the COVID-19 pandemic on the transport sector and travel behavior in the rural peripheries. They utilized the results of the aforementioned ITF [8] works and additional literature to understand how COVID-19 has affected rural mobility, to gain implications for how to plan for sustainable rural mobility in the post-COVID world, and to understand the longer-term impacts of COVID-19. Despite negative short-term impacts of the pandemic in rural areas, they identified opportunities for changes in mobility behavior in the post-COVID era. Together with public transport, community-led transport services will potentially be strengthened as an essential “lifeline” in rural areas. Demand-responsive forms of rural mobility services and solutions, including but not limited to taxis and ride-hailing, will potentially gain more attentions in addressing mobility needs which has been covered by private cars. They consider the digital alternatives as a way to manage travel demands in rural areas, allowing goods and service delivered rather than individuals traveling for long distances. They also consider working-from-home as a potential for rural areas if this can stimulate migration to rural areas.

The pandemic is a kind of “wildcard” event, which has low probability of occurrence but high impacts on the society. Tori et al. [8] developed a participatory methodology for developing mobility visions with such embedded wildcard events and tested them it in a Belgian village to design mobility scenarios for the village for 2050. In the vision development process, they observed the primary role of wildcard events being an incentive to step out of participant’s comfort zones to think more freely and openly. As the authors imply in the paper, it is more difficult in rural areas than in urban areas to think about mobility options flexibly and freely because of the dominance of the cars in everyday mobility and the unavailability of alternatives to cars. This could also potentially hinder participatory planning process because, for rural residents, business-as-usual deems to be an only available mobility option. In this sense, this research opens up a potential method to address this problem underlying in rural area, while further developments will be needed to establish it as a part of the participatory approach in rural area for sustainable mobility.

Agriesti et al. [1] analyze the social, technological, economic, environmental and policy challenges faced by rural areas and the applicability of available innovative solutions for rural areas to address these identified challenges. Their analysis results, based on surveys and workshops with Estonian municipalities, highlights that no single innovative solution will be able to address all challenges that low-density area faces. There are various hinderance factors in social, technological, economic, environment and policy domains to implement innovative mobility solutions. They also identify an “policy void” in this domain, which leads to less or hardly existent support to overcome such hinderances.

Poltimäe et al. [8] confirms the conclusion by Agriesti et al. [1] as they state “single novel mobility solutions are seldom applicable for all rural travellers” in their extensive literature review onto innovative mobility solutions in rural areas. They clearly recognize the issues of research gaps between everyday mobility of rural inhabitants and mobility needs of visitors, such as tourists and owners of second homes. The ten theses that they discuss in the paper will potentially serve as an important guidance for future research onto the topic of rural mobility to fill this research gap.

Hrelja et al. [5] present their three cases studies in Sweden onto transit-oriented development (TOD) in low-density and peri-urban contexts. Their key finding is that enablers and barriers for TODs in the low-density contexts are largely similar to the ones in cities. Their analysis implies that TOD may work also in low-density peri-urban regions as a potential mid- to long-term strategy to overcome the general difficulty of public transport in such regions, but only when the value of real estate property is increased and attractive living conditions in close proximity of urban areas can be offered.

Three papers in this TC focus on specific modes of transport or planning approaches in rural contexts. Some of them could be contextualized as innovative mobility solutions, but largely they are based on the classical means of transport. Hansson et al. [3] analyze the wider effects on overall railway patronage arising from filling interval gaps of local railway services in off-peak hours. They analyzed the effect on public transport usage in four cases in southern Sweden, where at least hourly all-day rail or bus services were newly introduced, filling interval gaps. Such additional services address the difficulties of rural public transport as pointed out by Poltimäe et al. [8] that public transport supply it is often not flexible enough to respond to the diverse travel needs of the users. Hansson et al. [3] demonstrate that such improved coverage of longer hours leads to overall patronage growth throughout the day. This is an important addition to the knowledge to understand the benefit of systematic and regular public transport services in rural contexts, which would enhance the opportunities of its use and results in higher ridership.

Heinitz [4] tested his assessment method for shared and on-demand mobility services in rural areas. With his framework, he assessed two scenarios about regulatory options, one being that ride-sourcing services do not directly compete against public transport and serve as a complimentary service to it, and the other being that sharing of rides are liberalized and compete against public transport while incentivized to shift from single-person car use. With a case study in Germany, he estimates the ranges of revenues, extra vehicle kilometres, and necessary public budgets.

Scappini et al. [8] presents their approach for a regional bike network on the island of Sardinia, Italy. Their planning results are of course local to the island’s context, but more importantly they demonstrate how a systematized planning approach for regional bicycle network could look like, as well as how to estimate potentials of cycling in a regional context where cycling has not played a significant role in the transport system.

The wide range of research outcomes are included in this Topical Collection. At the same time, many of them pose further research questions that will have to be addressed in the future. Although rural mobility is gaining more attention in policy-making and scholarly research, it is still a far less studied domain especially when it is compared to the urban counterparts. How can policy goals for rural mobility in light of both accessibility and sustainability look like? What kind of mobility needs will have to be prioritized to be covered publicly by existing and emerging mobility services to deliver sustainable mobility in rural contexts, and what else may have to remain covered by cars? How can transport policy instruments be effectively implemented in rural areas? It is also important to address how the digital transformation of the society will change rural mobility needs, too. Despite the ongoing agglomeration to urban areas, rural areas will continue to accommodate a significant number of people, industries, and facilities. Therefore, major research efforts will be required in the future to make rural mobility sustainable.
